# Internal Stress Prediction and Measurement of Mid-Infrared Multilayer Thin Films

**DOI:** 10.3390/ma14051101

**Published:** 2021-02-26

**Authors:** Chuen-Lin Tien, Kuan-Po Chen, Hong-Yi Lin

**Affiliations:** 1 Department of Electrical Engineering, Feng Chia University, Taichung 40724, Taiwan; chaioli0921@gmail.com; 2 Electrical and Communications Engineering, Feng Chia University, Taichung 40724, Taiwan; bq100035@hust.edu.tw

**Keywords:** internal stress, interfacial force, multilayer thin film, band-pass filter

## Abstract

We present an experimental method for evaluating interfacial force per width and predicting internal stress in mid-infrared band-pass filters (MIR-BPF). The interfacial force per width between the two kinds of thin-film materials was obtained by experimental measurement values, and the residual stress of the multilayer thin films was predicted by the modified Ennos formula. A dual electron beam evaporation system combined with ion-assisted deposition was used to fabricate mid-infrared band-pass filters. The interfacial forces per width for Ge/SiO_2_ and SiO_2_/Ge were 124.9 N/m and 127.6 N/m, respectively. The difference between the measured stress and predicted stress in the 23-layer MIR-BPF was below 0.059 GPa. The residual stresses of the four-layer film, as well as the 20-layer and 23-layer mid-infrared band-pass filter, were predicted by adding the interface stress to the modified Ennos formula. In the four-layer film, the difference between the predicted value and the measured stress of the HL (high–low refractive index) and LH (low–high refractive index) stacks were −0.384 GPa for (HL)^2^ and −0.436 GPa for (LH)^2^, respectively. The predicted stress and the measured stress of the 20-layer mid-infrared filter were −0.316 GPa and −0.250 GPa. The predicted stress and the measured stress of the 23-layer mid-infrared filter were −0.257 GPa and −0.198 GPa, respectively.

## 1. Introduction

Thin-film coatings are an important part of infrared devices, and usually must have good performance in a wide spectral range. For this reason, even a small improvement of the infrared thin-film coatings can significantly improve the performance of the infrared system, especially in the control of residual stress, which will have a significant impact. The problem of residual stress in multilayer coatings is related to the number of layers, thin-film thickness, layer and substrate materials, deposition technique, process parameters, and working pressure etc. Residual stress is a major threat to thin-film components. Larger residual stress may cause film cracking. It is necessary to control the internal stress in thin films. The residual stress in a thin film coating is commonly controlled by tuning the process conditions. However, there are few ways to control the residual stress in multilayer coatings. In this work, an efficient method for controlling and predicting residual stress in multilayer thin films was studied.

In 2005, Shao et al. [1] studied the residual stress in (ZrO_2_/SiO_2_)^x^ multilayer films, in which the ZrO_2_ film was under tensile stress and the SiO_2_ film under compressive stress. Since the residual stress states of the two thin films were different, the proper number of layers could compensate for the multilayer residual stress. When the number of cycles was nine, the lowest compressive stress of 76 MPa could be obtained. Oliver et al. [2] added a compressive Al_2_O_3_ film into HfO_2_/SiO_2_ multilayers with a total film thickness of 5 μm. Compensating tensile stress above 80 MPa to compressive stress below 70 MPa. In the same year, Li et al. [3] used a multi-beam optical stress sensor (MOSS) to measure the residual stress evolution during the deposition of HfO_2_/SiO_2_ multilayer films. The multilayer films could be controlled by adjusting the thickness of each layer. Although the residual stress could be adjusted in real-time, it was difficult to achieve optical performance.

In 2014, Lemarquis et al. [4] proposed a multilayer film stress model that assumed the multilayer residual stress was the product of the single-layer residual stress and the film thickness. After measuring the residual stress of the multilayer film deposited on one side of the substrate, a multilayer film of the same residual stress was deposited on the other side of the same substrate. In this particular case, the residual stresses generated on both sides of the substrate canceled each other out. In 2017, Begou et al. [5] proposed an accurate model based on the Stoney formula that could extract the deformation caused by the residual stress after a film was deposited. This model was used to determine the deformation caused by the residual stress in a multilayer film. In order to accurately predict the flatness of the component, they pointed out that different film materials and process technologies affect the film’s residual stress and deformation. In 2018, Probst et al. [6] applied counter-balancing coating method and achieved good residual stress compensation for Cr/Ir multilayer coatings based on using numerical methods to find the change between stress and film thickness. Their results indicated that the stress in the Cr/Ir multilayers could be reduced by adjusting the thickness of the Cr film. They also adapted this method to deposit SiO_2_ films on the back surface. The sag of a coated glass substrate with a 25 mm diameter and a 1.07 ± 0.2 mm thickness was under 38 nm. In 2019, Begou et al. [7] improved the experimental method. After considering the deformation of the thin film and the substrate caused by film stress, a portion of the residual stress was offset by depositing a multilayer film on the back of the substrate. They successfully achieved stress compensation on a 24 mm × 26 mm substrate and reduced the average sag of the bandpass filter to −16.73 ± 3 nm. They reported that the deformation of the substrate is caused by the coating on the front surface of the substrate. The residual stress of the back surface coating needs to be compensated. Furthermore, the multilayer films deposited on the back also affect the spectral properties. Therefore, it is difficult to meet the requirements of spectral properties and low residual stress of double-sided coating at the same time. Liu et al. [8] composed a multilayer anti-reflection film with tensile residual stress for Al_2_O_3_ thin film and compressive stress for SiO_2_ thin film. The two opposite residual stresses were reduced after alternating deposition. When the residual stress of two materials is opposite, but the product of residual stress and thickness is close, they can cancel each other.

In 2020, Oliver et al. [9] proposed a non-uniform deposition method for SiO_2_ thin film to compensate for the stress in 3.3 µm-thick multilayer films deposited on a fused silica substrate with a diameter of 100 mm and a thickness of 3 mm. In a non-uniformly deposited SiO_2_ film, the thickness of the substrate was thinner at the center and the periphery was thicker, which made the SiO_2_ film to be in a state of tensile residual stress. This tensile state can be used to reduce the compressive residual stress of the multilayer film. However, in order to accurately evaluate the film thickness distribution on the substrate, it is necessary to understand the coating geometry, including the positions of the sample holder, the evaporation source, and the film thickness monitoring system. Most of the above scholars studied the influence of a single-layer film on the residual stress of a multilayer film; however, they paid little attention to the interfacial force per unit width between two adjacent films which affect the residual stress of the multilayer films.

Due to the complexity of multilayer coating parameters, such as materials properties, thickness variation, interfacial roughness, surface diffusion and deposition process, reducing the multilayer coating stress is always difficult and interesting. Therefore, we present a stress prediction model to estimate the multilayer residual stress. By fabricating two kinds of four-layer-stacking films were used for an interfacial stress evaluation. In this work, we focused on internal stress prediction and control in the mid-infrared band-pass filters (BPFs). Because optical interference filters are composed of two or more thin-film materials, and the interfacial forces per unit width between two or more materials are different, it is possible to control residual stress by alternating the coating parameters. To achieve this goal, the internal stress prediction of multilayer films is helpful for residual stress control.

In the present work, we prepare two kinds of mid-infrared band-pass filters (MIR-BPF), paying attention to compare the multilayer stress prediction and internal stress measurement. A modified Ennos formula was proposed to analyze and predict the interfacial and internal stresses of multilayer thin films with high- and low- refractive index materials. Corresponding experiments were carried out to demonstrate the performance of the modified Ennos formula.

## 2. Materials and Methods

The most common method to study residual stress in the thin film is to measure the coating sample curvature before and after film deposition. The Stoney formula [10] is widely used in thin-film residual stress calculations. It assumes that the substrate thickness should be much larger than that of the films. The original Stoney equation was only valid for thin films with uniaxial stress on the elastically isotropic substrates. Since residual stress in a thin film is typically biaxial and not uniaxial distribution [11]. The residual stress of thin film was inferred from the change in the curvature of the substrate caused by the stressed film. Because the state of deformation is equi-biaxial strain at each point of the substrate, the resistance to deformation can be represented in terms of the biaxial elastic modulus of the substrate materials, which is denoted by E_s_/(1 − υ_s_). Thus biaxial stress components are taken into account. The modified Stoney formula is expressed as follows:

(1) $$\sigma \;=\;\frac{E_{s}t_{s}^{2}}{6\left(1-\nu_{s}\right)t_{f}}\left(\frac{1}{R}-\frac{1}{R_{0}}\right),$$

where σ is the residual stress in thin films. t_f_ is the thickness of the film, R_0_ and R correspond to the radius of the curvature of the substrate measured before and after film deposition. For a given substrate, Young’s modulus (E_s_), Poisson’s ratio (υ_s_), and thickness (t_s_) are constants. By convention, σ is negative for compressive stress and positive for tensile stress.

In general, thin-film internal stress prediction requires a suitable model, which can also assist in comprehending the internal stress magnitude within multilayer coatings. In the internal stress prediction, the magnitude of the internal stress value also depends on the characteristics of the thin film and the substrate, such as the coefficient of thermal expansion, Young’s modulus, and the Poisson ratio. However, it is difficult to find the specific parameter values involved in the model. A simple formula of internal stress prediction in multilayers was proposed by Ennos [12], who stated that the internal stress in multilayer films can be given by the weighted average of each internal stress in a single-layer film. The Ennos formula is expressed as follows:

(2) $$\sigma_{\mathrm{avg}}=\overset{n}{\underset{i=1}{\sum }}\left(\sigma_{\mathrm{Hi}}t_{\mathrm{Hi}}+\sigma_{\mathrm{Li}}t_{\mathrm{Li}}\right)/(t_{\mathrm{Hi}}+t_{\mathrm{Li}}),$$

where σ_avg_ is the average internal stress of the multilayer film, σ_Hi_ and σ_Li_ are the internal stress values of the high and low refractive indices, and t_Hi_ and t_Li_ are the film thickness values of the high and low refractive indices.

By measuring the internal stress in single-layer films, the internal stress in multilayer films can be predicted. The Ennos formula’s accuracy of film stress prediction was discussed by Guo et al. [13], who had pointed out that the internal stress in even numbers of multilayers will be close to a constant value for the alternating multilayer films. However, this may not be consistent with the actual stress value because the interface force effect is not included in the Ennos formula.

Janssen [14] made an argument that the internal stress of multilayer films can be divided into two parts. One is the sum of the forces of various materials. The other is the product of the interfacial force and the number of interfaces. The internal stress associated with the interfaces may contribute to the overall internal stress in multilayers. With this idea, the Ennos formula can be modified as follows:

(3) $$\sigma_{\mathrm{avg}}=\overset{n}{\underset{i=1}{\sum }}\left(\sigma_{\mathrm{Hi}}t_{\mathrm{Hi}}+\sigma_{\mathrm{Li}}t_{\mathrm{Li}}\right)/(t_{\mathrm{Hi}}+t_{\mathrm{Li}})+(m\;f_{\mathrm{interface}})/(t_{\mathrm{Hi}}+t_{\mathrm{Li}}),$$

where m is the number of interfaces and f_interface_ is the interface force.

The interface stress has been studied extensively. In 1993, Ruud et al. [15] found that the internal stress in Ag/Ni multilayer films was not equal to the sum of the internal stresses of the single-layer films. This result showed that the internal stress in multilayer films is complicated. It is not simply obtained by adding the stress values of single-layer films but rather is affected by the interfacial stress. They proposed a method to measure the interface force and carried out the experiment with the X-ray diffraction method. The experimental value of the Ag/Ni interfacial force was −2.21 ± 0.67 J/m^2^ and the theoretical value is 0.32 J/m^2^. The theoretical value is quite different from the experimental value. It is not easy to measure the elastic constants of multilayer films accurately.

In 2000, Spaepen [16] improved on the experimental method by using optical interferometry to measure the change in curvature of multilayer films. The thin-film internal stress could be calculated by the radius of curvature before and after coatings. It was found that interfacial stress significantly exists in multilayer films. Spaepen also proposed a method to evaluate the interfacial stress by the curvature method, and showed that the interfacial stress can be tensile or compressive. A number of studies have also used similar methods for the evaluation of interfacial force in multilayer films [17,18,19]. However, this method evaluates the average interfacial force of two materials. In some cases, if the number of multilayers is even (i.e., the number of interfaces is odd), the average interfacial force values may be incorrect.

**Figure 1:**
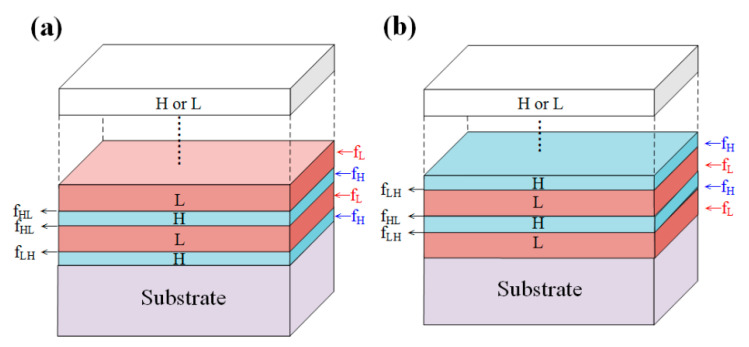
Interfacial force model for (**a**) (HL)^x^ periodic structure; (**b**) (LH)^x^ periodic structure.

In this study, we modified Spaepen’s method to evaluate the interfacial force per unit width. By four-layer coating experimental methods, the interfacial force between two adjacent films could be obtained. We combined the ideas of Janssen [14] and Spaepen [16] to modify the original Ennos formula and predict the residual stress in multilayer coatings. We assumed that a multilayer thin-film structure (HL)^x^ or (LH)^x^ is composed of two kinds of thin-film materials, in which the power *x* is the coating period or the number of cycles, H stands for high-index materials and L stands for low-refractive-index materials. It should be noted that the internal stress of both materials can be tensile or compressive and can be determined by the internal stress measurement. First, the deposition of the H material is completed and then the bilayer structure of the L material is deposited to form a high–low refractive index (HL) stack. Next, the surface of the L material will be covered by the H material. Finally, after the surface of the L material is formed, and the surface of the H material disappears, thus a four-layer (HL)^2^ stack is formed. Similarly, a four-layer (LH)^2^ stacking interface can also be formed. The interfacial stress model is shown in Figure 1. By subtracting the two times of bilayer film (Δ_HL_) from the force per unit length of the four-layer film (Δ_HLHL_), the interface force (f_LH_) per unit width can be obtained from the following equation:

(4) $$\Delta_{\mathrm{HLHL}}\left(F/w\right)-2\Delta_{\mathrm{HL}}\left(F/w\right)={\;f}_{\mathrm{LH}},$$

where f_LH_ is the interfacial force of the low refractive index material deposited on the high refractive index material. Similarly, f_HL_ can be expressed as:

(5) $$\Delta_{\mathrm{LHLH}}\left(F/w\right)-2\Delta_{\mathrm{LH}}\left(F/w\right)={\;f}_{\mathrm{HL}},$$

where f_HL_ is the interfacial force of the high refractive index material deposited on the low refractive index material. The Ennos formula is incomplete and does not consider the effect of interface force per unit width on the residual stress in multilayer coatings. Therefore, the internal stress in multilayer thin films should include not only the internal stress of each single-layer film, but also the interface force per unit width and the number of interfaces. In the interfacial force model, the f_HL_ and f_LH_ interface forces per unit width are multiplied by the number of interfaces and added to the Ennos formula for the internal stress correction. Here f_HL_ and f_LH_ have a different number of interfaces when the number of coating layers is odd or even, therefore, the number of coating layers should be considered. If considering odd and even numbers of multilayer coatings, the modified stress prediction formulas are given as follows:

For the odd-numbered multilayer stacks:

(6) $$\sigma_{1,3,\ldots 2n-1}=\frac{\sigma_{f1}\cdot t_{f1}+\sigma_{f2}\cdot t_{f2}++\sigma_{\mathrm{fn}}\cdot t_{\mathrm{fn}}}{t_{f1}+t_{f2}+\ldots +t_{\mathrm{fn}}}+\frac{\left(\frac{n-1}{2}\right)f_{\mathrm{HL}}+\left(\frac{n-1}{2}\right)f_{\mathrm{LH}}}{t_{f1}+t_{f2}+\ldots +t_{\mathrm{fn}}}=\frac{\sum_{i}^{n}\sigma_{\mathrm{fi}}\cdot t_{\mathrm{fi}}}{\sum_{i}^{n}t_{\mathrm{fi}}}+\frac{\left(\frac{n-1}{2}\right)f_{\mathrm{HL}}+\left(\frac{n-1}{2}\right)f_{\mathrm{LH}}}{\sum_{i}^{n}t_{\mathrm{fi}}}$$

For predicting stress in odd-number coating layers, the interface numbers of both f_HL_ and f_LH_ are equal to $\frac{n-1}{2}$. On the contrary, in order to predict stress in even-numbered coating layers, the interface numbers of both f_HL_ and f_LH_ are different and depend on the different structures of (HL)^x^ and (LH)^x^ periodic stacks. Here x is the periodic number of HL and low–high refractive index (LH) stacks. Therefore, in the even-numbered coating stress estimation, the following two situations should be considered.

For the even-numbered stacks with (HL)^x^ periodic structure:

(7) $$\sigma_{2,\ldots 2n}=\frac{\sigma_{f1}\cdot t_{f1}+\sigma_{f2}\cdot t_{f2}+\ldots +\sigma_{\mathrm{fn}}\cdot t_{\mathrm{fn}}}{t_{f1}+t_{f2}+\ldots +t_{\mathrm{fn}}}+\frac{\left(\frac{n}{2}-1\right)f_{\mathrm{HL}}+\left(\frac{n}{2}\right)f_{\mathrm{LH}}}{t_{f1}+t_{f2}+\ldots +t_{\mathrm{fn}}}=\frac{\sum_{i}^{n}\sigma_{\mathrm{fi}}\cdot t_{\mathrm{fi}}}{\sum_{i}^{n}t_{\mathrm{fi}}}+\frac{\left(\frac{n}{2}-1\right)f_{\mathrm{HL}}+\left(\frac{n}{2}\right)f_{\mathrm{LH}}}{\sum_{i}^{n}t_{\mathrm{fi}}}$$

For the even-numbered stacks with (LH)^x^ periodic structure:

(8) $$\sigma_{2,\ldots 2n}=\frac{\sigma_{f1}\cdot t_{f1}+\sigma_{f2}\cdot t_{f2}+\ldots +\sigma_{\mathrm{fn}}\cdot t_{\mathrm{fn}}}{t_{f1}+t_{f2}+\ldots +t_{\mathrm{fn}}}+\frac{\left(\frac{n}{2}\right)f_{\mathrm{HL}}+\left(\frac{n}{2}-1\right)f_{\mathrm{LH}}}{t_{f1}+t_{f2}+\ldots +t_{\mathrm{fn}}}=\frac{\sum_{i}^{n}\sigma_{\mathrm{fi}}\cdot t_{\mathrm{fi}}}{\sum_{i}^{n}t_{\mathrm{fi}}}+\frac{\left(\frac{n}{2}\right)f_{\mathrm{HL}}+\left(\frac{n}{2}-1\right)f_{\mathrm{LH}}}{\sum_{i}^{n}t_{\mathrm{fi}}}$$

## 3. Experiments

### 3.1. Thin Film Fabrication

In this study, (SiO_2_/Ge)^11^/SiO_2_ and (SiO_2_/Ge)^10^ mid-infrared band-pass filter (MIR-BPF) films were deposited on silicon substrates using a dual electron beam evaporation system (SGC-22SA, Showa Shinku, Sagamihara, Kanagawa, Japan) combined with end-Hall type ion-assisted deposition (Mark II+, Veeco Co., Plainview, NY, USA). The deposition temperature was kept at 150 °C. The control of the film thickness in each of the layers was carried out using quartz monitoring (CRTM-6000M, ULVAC Inc., Chigasaki, Kanagawa, Japan.) and a reflective optical monitoring system (SOCS-1α, Showa Shinku, Sagamihara, Kanagawa, Japan). The pressure of the vacuum chamber was kept below 1 × 10^−3^ Pa. The electron beam power was 3.5 kW. The anode voltage and current used for ion-assisted deposition was 150 V and 3 A, respectively. The Ar flow rate for the ion source was 13 sccm.

### 3.2. Thin Film Measurements

In this study, the thin film stress measurement system utilized a home-made Twyman-Green interferometer to evaluate the internal stress in thin films. A self-developed stress analysis program based on fast Fourier transform (FFT) [20,21] was used for the internal stress measurement. After a thin film is coated on the substrate, the surface of a circular glass substrate deforms, and its microscopic surface contour becomes a bowl-shaped curved surface. Surface deformation of the substrate is entirely the result of the residual stresses present in the thin films. The curved surface is regarded as part of a spherical surface, and the radius of curvature can be fitted. Using the modified Stoney formula, the internal stress in the thin films was calculated from the radius change of the curvature of the substrate before and after coatings. The surface roughness in these thin-film multilayers was measured by a Linnik microscopic interferometer associated with FFT and a Gaussian filter according to the method described in a previous publication [22]. The optical transmittance of the thin films was measured using a FTIR spectrometer (Frontier, PerkinElmer^®^ Inc., Waltham, MA, USA). The measurement range of the thin film transmittance versus different wavenumbers varied from 5000 to 650 cm^−1^. In addition, the microstructure of the films was examined by X-ray diffraction (XRD), using a SIEMENS D-5000 diffractometer (Siemens, Munich, Germany).

### 3.3. Optical Band-Pass Filter Design

**Figure 2:**
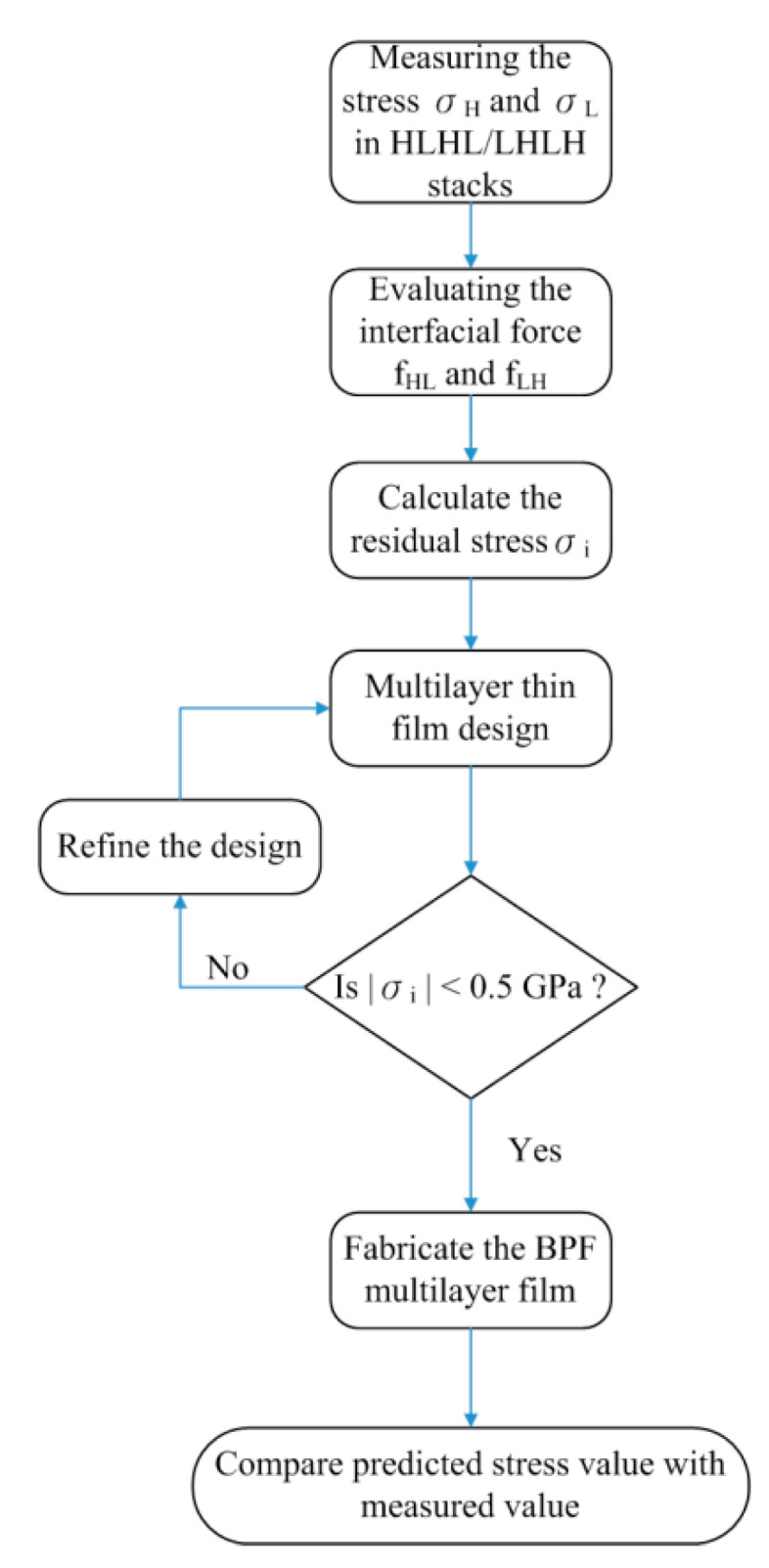
The flow chart of mid-infrared band-pass filters (MIR-BPFs) design.

We designed a low-stress MIR-BPF through the steps shown below. Figure 2 indicates the flow chart of the MIR-BPFs design. First, two types of MIR-BPFs were designed by Essential Macleod V10.1 software (Thin Film Center Inc., Tucson, AR, USA). The wide band-pass filter could be composed of the long-pass and low-pass filters [23], and germanium and silicon dioxide were used as the high and low refractive index materials, respectively. Second, single-layer Ge and SiO_2_ films were prepared and the internal stress in these films was measured by a Twyman-Green interferometer. In these prepared thin films, the film thicknesses were about 60 nm for Ge film and 190 nm for SiO_2_ film, respectively, corresponding to the quarter-wave optical thickness. Third, the interfacial forces were obtained using Equations (4) and (5). In order to apply these equations, it was also necessary to prepare two-layer films and four-layer films. The individual layer thickness in the stacks was also equal to the quarter-wave thickness. Fourth, using the film design, the single-layer film stress, and the interfacial force of two materials, the stress in the multilayer films was calculated by a self-developed MATLAB^®^ program. Through the MATLAB^®^ R2020b software (MathWorks, Natick, MA, USA), the product of the film thickness and stress in each layer was recorded, and the average stress, which varied with the number of layers, could be determined. This is helpful to the design and optimization of the optical band-pass filters. One of the research objectives is to fabricate optical filters with low internal stress. If the predicting internal stress value exceeded ±0.5 GPa, the multilayered film design needed to be improved. In this study, the internal stresses in the two single-layer films were compressive and the interfacial forces were tensile. In this case, increasing the film thickness and the number of coating layers could dramatically reduce the internal stress. We used these features to design optical band-pass filters that could meet optical performance needs while maintaining low internal stress. Finally, the designed optical transmittance and predicted stress value were compared with the experimental results.

## 4. Results and Discussion

**Figure 3:**
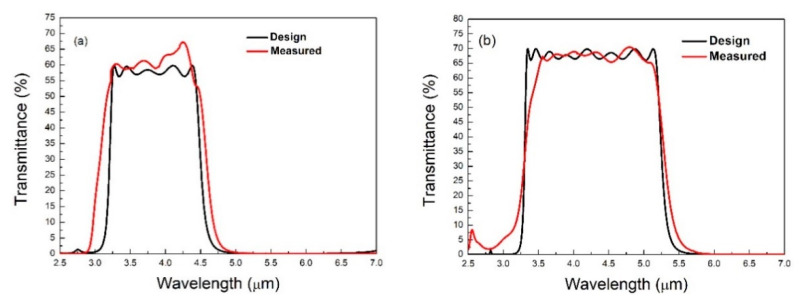
Transmission spectrum of (**a**) 20-layer; (**b**) 23-layer BPFs.

Figure 3a,b show the optical spectrum of a 20-layer and 23-layer MIR-BPF, respectively. The pass-band of the 20-layer BPF covered a wavelength of 3.4 to 4.3 μm, and the average transmittance of the pass-band was about 60%. The transmittance of the cut-off band was less than 1%, indicating a good cut-off effect. The measured and designed spectral curves of the 20-layer BPF were slightly different. This may have been due to the refractive index of the thin film not being constant in the multilayers and the density of the thin film being related to the refractive index. As shown in Figure 3b, the measurement and design transmittance curves of the 23-layer BPF were similar. The measurement spectra of the 23-layer BPF showed that the pass-band wavelength was from 3.3 μm to 5.5 μm, the average transmittance was about 65%, and the transmittance of the rejection band was below 1%.

**Figure 4:**
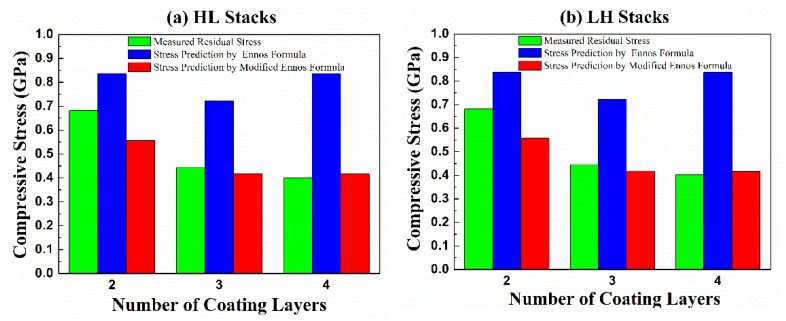
Comparison of the measured stress and the predicted stress from two-layer to four-layer films (**a**) high–low refractive index (HL) stack; (**b**) low–high refractive index (LH) stack.

The internal stress of the single-layer Ge and SiO_2_ films were −1.775 ± 0.275 GPa and −0.588 ± 0.039 GPa, respectively. The compressive stress of the Ge single-layer film was much larger than that of the SiO_2_ thin film. In the SiO_2_/Ge/SiO_2_/Ge and Ge/SiO_2_/Ge/SiO_2_ four-layer films, the measured stresses were −0.401 ± 0.013 GPa and −0.483 ± 0.016 GPa, respectively. However, the predicted stress values from the Ennos formula were −0.837 GPa for both film stacks, which were much larger than the measured values. It could be seen that interfacial stress existed in the structure of the multilayer films. By using Equations (4) and (5), two kinds of interfacial forces per unit width were found to be f_HL_ = 124.9 N/m and f_LH_ = 127.6 N/m, respectively, and both were identified as tensile interface forces. Figure 4 shows the predicted stresses of 2–4 layers with quarter-wave thickness of SiO_2_ and Ge thin films and compared with the measured stress. Figure 4a,b indicate the internal stress in the HL and LH stacks predicted by the modified Ennos formula and the original Ennos formula. The original Ennos formula does not consider the influence of the interface force, when the number of coating layers and interfaces is increased, there is a large deviation between the predicted value of the Ennos formula and the measured value. For example, in the SiO_2_/Ge/SiO_2_/Ge and Ge/SiO_2_/Ge/SiO_2_ four-layer films, the internal stress differences between the stress values predicted by the Ennos formula (without considering the interfacial force) and the measured stresses were 0.354 GPa and 0.436 GPa, respectively. On the contrary, the differences between the predicted value (with the interfacial force evaluation) and the measured stress were 0.067 GPa and 0.015 GPa, respectively. These results showed that the internal stress of the four-layer film was predicted to be closer to the measured value by the modified Ennos formula with the interfacial force evaluation.

**Figure 5:**
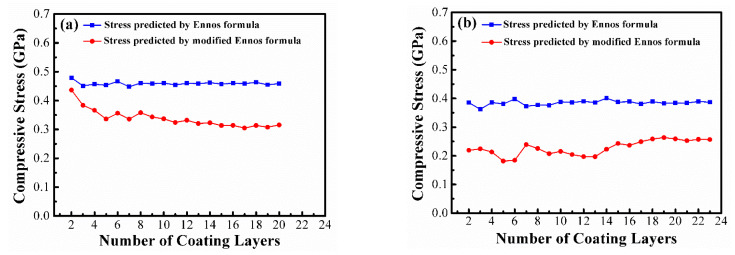
Internal stress prediction and comparison of (**a**) 20-layer; (**b**) 23-layer MIR-BPFs.

In order to verify whether the modified Ennos formula could be applied to the multilayer films, 20-layer and 23-layer MIR-BPF films were prepared using the same process parameters and measured by a Twyman-Green interferometer. Figure 5 shows the predicted stresses based on the 20-layer and 23-layer non-quarter wave thickness designs composed of SiO_2_ and Ge thin films. In Figure 5, the blue square indicates that the original Ennos formula and MATLAB^®^ numerical program are used to simulate and calculate the residual stress of multilayer film with different coating layers, while the red solid circle represents the use of the modified Ennos formula and the MATLAB^®^ numerical program to simulate and predict the residual stress of the multilayer film with different coating layers. The predicted stress values in the 20-layer and 23-layer MIR-BPFs were −0.316 GPa and −0.257 GPa, respectively, and the measured stresses in the 20-layer and 23-layer MIR-BPFs were −0.250 GPa and −0.198 GPa, as shown in Figure 5a,b, respectively. The results show that the predicted value of the residual stress of the MIR-BPF multilayer film was different from the actual measured value. The difference between the measured and predicted values was 0.066 GPa for 20-layer and 0.059 GPa for 23-layer MIR-BPF, as indicated in Table 1. The change in the radius of curvature before and after the multilayer coatings was different, the data could be calculated from Table 1 that the deformation of the 23-layer BPF multilayer film with a thickness of 10.585 μm was smaller than that of the 20-layer BPF with a thickness of 8.306 μm. The residual stress measurement has the error of about ± 5%. The multilayer thermal stress and the interfacial stresses of film-substrate were ignored in the proposed prediction model. These reasons might cause a deviation of 20–25% between the predicted and measured stresses in this work. On the other hand, the compressive internal stress in the 23-layer MIR-BPF was lower than that in the 20-layer MIR-BPF, due to the increase in the number of interfaces and the significant effect of the interfacial tensile force on the structure of the film layer, which could balance the compressive internal stress. In this case, increasing the number of coating layers and the total film thickness can help to reduce the compressive stress in the multilayer films. However, there is a large difference in residual stress between the two kinds of thin films, and many coating layers may be needed to significantly reduce the internal stress due to the large internal stress contribution of Ge films.

**Figure 6:**
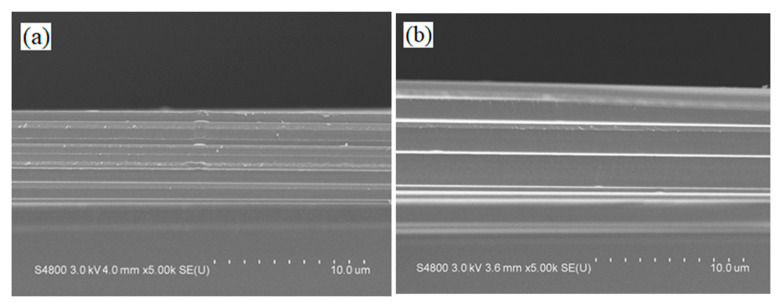
SEM cross-section image of (**a**) 20-layer; (**b**) 23-layer BPFs.

**Figure 7:**
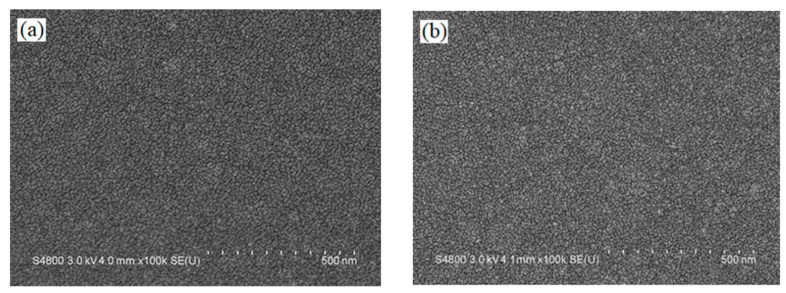
The top-view SEM image of (**a**) 20-layer; (**b**) 23-layer BPFs.

**Figure 8:**
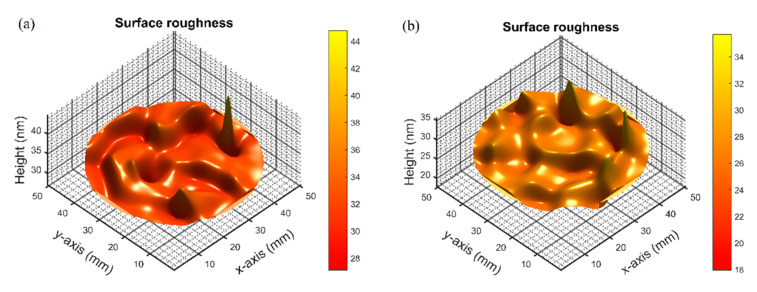
The surface roughness of (**a**) 20-layer; (**b**) 23-layer BPFs.

**Figure 9:**
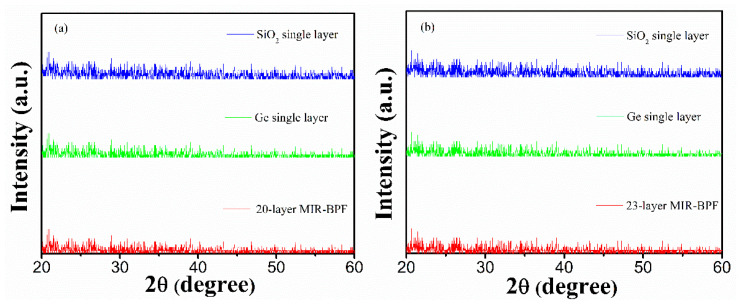
XRD patterns of (**a**) 20-layer; (**b**) 23-layer BPFs.

Figure 6a,b show cross-sectional SEM images of the 20-layer and 23-layer MIR-BPFs. It could be observed from the SEM images that there were some film interfaces. These interfaces showed the presence of forces between the layers. The dark zone under the multilayer is the silicon substrate. Thus, the multilayer periods are clearly evidenced. It could also be observed that the film structure was dense and non-columnar. The SEM surface images of the 20-layer and 23-layer MIR-BPFs are shown in Figure 7a,b. The surface of these multilayer film deposits appeared to be smooth. The surface roughness of the MIR-BPF measured by the home-made Linnik microscopic interferometer is shown in Figure 8a,b. The room-mean-square (RMS) surface roughness was 1.1 nm and 1.3 nm, respectively. The low scattering loss could be obtained a high transmittance in the passband. Figure 9a,b shows the X-ray diffraction (XRD) patterns of 20-layer and 23-layer MIR-BPFs, respectively. Since the multilayer coatings were grown at a heating temperature of 150 °C. According to X-ray diffraction measurements, the diffraction pattern consisted of a diffuse-scattering curve, and there was no obvious diffraction peak. The results show that the single-layer SiO_2_ and Ge thin films have an amorphous-like structure. Similarly, the 20- and 23-layer MIR-BPFs also exhibit an amorphous structure.

## 5. Conclusions

This work deals with predication and controlling of internal stress in multilayered mid-infrared band-pass filters (MIR-BPFs). We proposed a method for evaluating interfacial force and predicting internal stress in two kinds of MIR-BPFs. The proposed method can be used as a reference for multilayer design to optimize the structure with a target of minimum stress. The interfacial force per unit width of the germanium and silicon dioxide films (f_HL_ and f_LH_) were 124.9 N/m and 127.6 N/m, respectively. The predicted stress values based on the interfacial force evaluation in the multilayer films are close to the measured values. The deviation between the measured stress and the predicted stress is 0.066 GPa for 20-layer and 0.059 GPa for 23-layer BPFs. The experimental results show that the proposed approach provides the multilayer coating stress prediction more accurately than the original Ennos formula. The optical transmittance measurement results show that it met the optical coating design result. The SEM images and XRD patterns show that the film’s surface morphology and microstructure were smooth and amorphous structure. The RMS surface roughness was below 1.3 nm, and showed the smooth surface of the optical band-pass filters.

## Figures and Tables

**Table 1:** Comparison of the predicted and measured stresses in 20-layer and 23-layer MIR-BPFs.

BPF Samples	Thickness (μm)	R_0_ (Before Coating)	R_1_ (After Coating)	Predicted Stress	Measured Stress	Stress Difference
20-Layer	8.306	−127.797 m	−25.488 m	−0.316 GPa	−0.250 GPa	0.066 GPa
23-Layer	10.585	−111.798 m	−23.171 m	−0.257 GPa	−0.198 GPa	0.059 GPa

## Data Availability

Data sharing not applicable.

## References

[1] Shao S.Y., Fan Z.X., Shao J.D. (2005). Influences of the period of repeating thickness on the stress of alternative high and low refractivity ZrO_2_/SiO_2_ multilayers. Acta Phys. Sin..

[2] Oliver J.B., Kupinski P., Rigatti A.L., Schmid A.W., Lambropoulos J.C., Papernov S., Kozlov A., Smith C., Hand R.D. (2012). Stress compensation in hafnia/silica optical coatings by inclusion of alumina layers. Opt. Express.

[3] Li J., Fang M., Hongbo H., Shao J., Fan Z., Li Z. (2012). Growth stress evolution in HfO_2_/SiO_2_ multilayers. Thin Solid Film.

[4] Lemarquis F. (2014). A thermal compensation of the stress-induced surface deflection of optical coatings using iso-admittance layers. Appl. Opt..

[5] Begou T., Lumeau J. (2017). Accurate analysis of mechanical stress in dielectric multilayers. Opt. Lett..

[6] Probst A.C., Begou T., Döhring T., Zeising S., Stollenwerk M., Stadtmüller J., Emmerich F., Lumeau J. (2018). Coating stress analysis and compensation for iridium-based x-ray mirrors. Appl. Opt..

[7] Begou T., Lemarchand F., Lemarquis F., Moreau A., Lumeau J. (2019). High-performance thin-film optical filters with stress compensation. J. Opt. Soc. Am. A.

[8] Liu H., Jensen L., Ma P., Ristau D. (2019). Stress compensated anti-reflection coating for high power laser deposited with IBS SiO_2_ and ALD Al_2_O_3_. Appl. Surf. Sci..

[9] Oliver J.B., Spaulding J., Charles B. (2020). Stress compensation by deposition of a nonuniform corrective coating. Appl. Opt..

[10] Stoney G.G. (1909). The tension of metallic films deposited by electrolysis. Proc. R. Soc. Lond. Ser. A.

[11] Grégory A., Eric C., Jozef K., Marco S., Gregory B.T., Etienne B., Gary L.D., Conal E.M., Chris H.S., Ludvik M. (2018). Stress in thin films and coatings: Current status, challenges, and prospects. J. Vac. Sci. Tech. A.

[12] Ennos A.E. (1966). Stresses Developed in Optical Film Coatings. Appl. Opt..

[13] Guo C.Q., Pei Z.L., Fan D., Liu R.D., Gong J., Sun C. (2016). Predicting multilayer film’s residual stress from its monolayers. Mater. Des..

[14] Janssen G.C.A.M. (2007). Stress and strain in polycrystalline thin films. Thin Solid Film.

[15] Ruud J.A., Witvrouw A., Spaepen F. (1993). Bulk and interface stresses in Ag/Ni multilayered thin films. J. Appl. Phys..

[16] Spaepen F. (2000). Interfaces and Stresses in Thin Films. Acta Mater..

[17] Josell D., Bonevich J.E., Shao I., Cammarata R.C. (1999). Measuring the interface stress: Silver/nickel interfaces. J. Mater. Res..

[18] Schweitz K.O., Bottiger J., Chevallier J., Feidenhansl R., Nielsen M.M., Rasmussen F.B. (2000). Interface stress in Au/Ni multilayers. J. Appl. Phys..

[19] Misra A., Kung H., Mitchell T.E., Nastasi M. (2000). Residual stresses in polycrystalline Cu/Cr multilayered thin films. J. Mater. Res..

[20] Tien C.L., Zeng H.D. (2010). Measuring residual stress of anisotropic thin film by fast Fourier transform. Opt. Express.

[21] Takeda M., Ina H., Kobayashi S. (1982). Fourier-transform method of fringe-pattern analysis for computer-based topography and interferometry. Appl. Opt..

[22] Tien C.L., Yu K.C., Tsai T.Y., Lin C.S., Li C.Y. (2014). Measurement of surface roughness of thin films by a hybrid interference microscope with different phase algorithms. Appl. Opt..

[23] Gaĭnutdinov I.S., Shuvalov N.Y., Sabirov R.S., Ivanov V.A., Gareev R.R., Mirkhanov N.G. (2009). Antireflection coatings on germanium and silicon substrates in the 3-5-μm and 8-12-μm windows of IR transparency. J. Opt. Technol..

